# Diagnostic Pitfalls of External Auditory Canal Cholesteatoma: Insights From a Case Report

**DOI:** 10.7759/cureus.81200

**Published:** 2025-03-25

**Authors:** Farid Syamil Ramli, Zara Nasseri, Asma Abdullah, Mohd. Hafiz Johari, Thean Yean Kew

**Affiliations:** 1 Otolaryngology–Head and Neck Surgery, Universiti Kebangsaan Malaysia Medical Centre, Kuala Lumpur, MYS; 2 Otolaryngology, Universiti Kebangsaan Malaysia Medical Centre, Kuala Lumpur, MYS; 3 Radiology, Universiti Kebangsaan Malaysia Medical Centre, Kuala Lumpur, MYS

**Keywords:** canal cholesteatoma, cholesteatoma, external auditory canal, external auditory canal cholesteatoma, otitis externa

## Abstract

External auditory canal cholesteatoma (EACC) is a rare occurrence characterized by the keratinized mass of squamous epithelial cells in the external ear canal, leading to bone erosion and potential damage to surrounding structures. It’s often misdiagnosed as otitis externa. Both conditions exhibit similar symptoms, which frequently result in misdiagnosis by clinicians. Though it is more common in older adults, EACC can occur in younger patients, as demonstrated in the case of an 18-year-old male. Insufficient diagnosis and delays in the management of this condition can result in significant complications. While the exact cause remains unclear, contributing factors may include canal trauma, chronic inflammation, or stenosis. We aim to share the experience of this pathology in order to highlight it as a differential diagnosis. This is particularly important in patients presenting with unresolved common ear symptoms.

## Introduction

External auditory canal cholesteatoma (EACC) is an infrequent otological disease, with a meta-analysis of clinical data over the last 30 years highlighting that canal cholesteatoma is often misdiagnosed as external otitis due to its rarity and the similarity of symptoms [1]. The prevalence varies, with an incidence of approximately 19.1% among those with external auditory canal (EAC) stenosis in patients with congenital aural atresia or stenosis [2]. Although this condition can manifest in individuals of any age group, it is more frequently observed in adults with a mean age of 41.3 years [3]. EACC is characterized by slow growth and may present with symptoms such as otorrhea, otalgia, ear fullness, or hearing loss or may be asymptomatic. The condition is marked by bone erosion and the accumulation of keratin material in squamous epithelial cells, primarily in the posteroinferior segment of the EAC [4].

The etiology and pathogenesis of EACC remain unclear; however, potential contributing factors may include canal trauma, prolonged inflammation of the ear canal, ear canal stenosis, or spontaneous development. Despite its slow growth and benign nature, EACC has the capacity to spread into local structures causing destruction of adjacent structures and loss of function. Diagnosis typically relies on clinical examination and radiological imaging, such as computed tomography (CT) scans, to evaluate the bony destruction and surgical planning [5].

We present a case of an 18-year-old individual with extensive EACC who was initially misdiagnosed as having otitis externa. The report explores potential causes, diagnostic considerations, and treatment modalities.

## Case presentation

**Figure 1:**
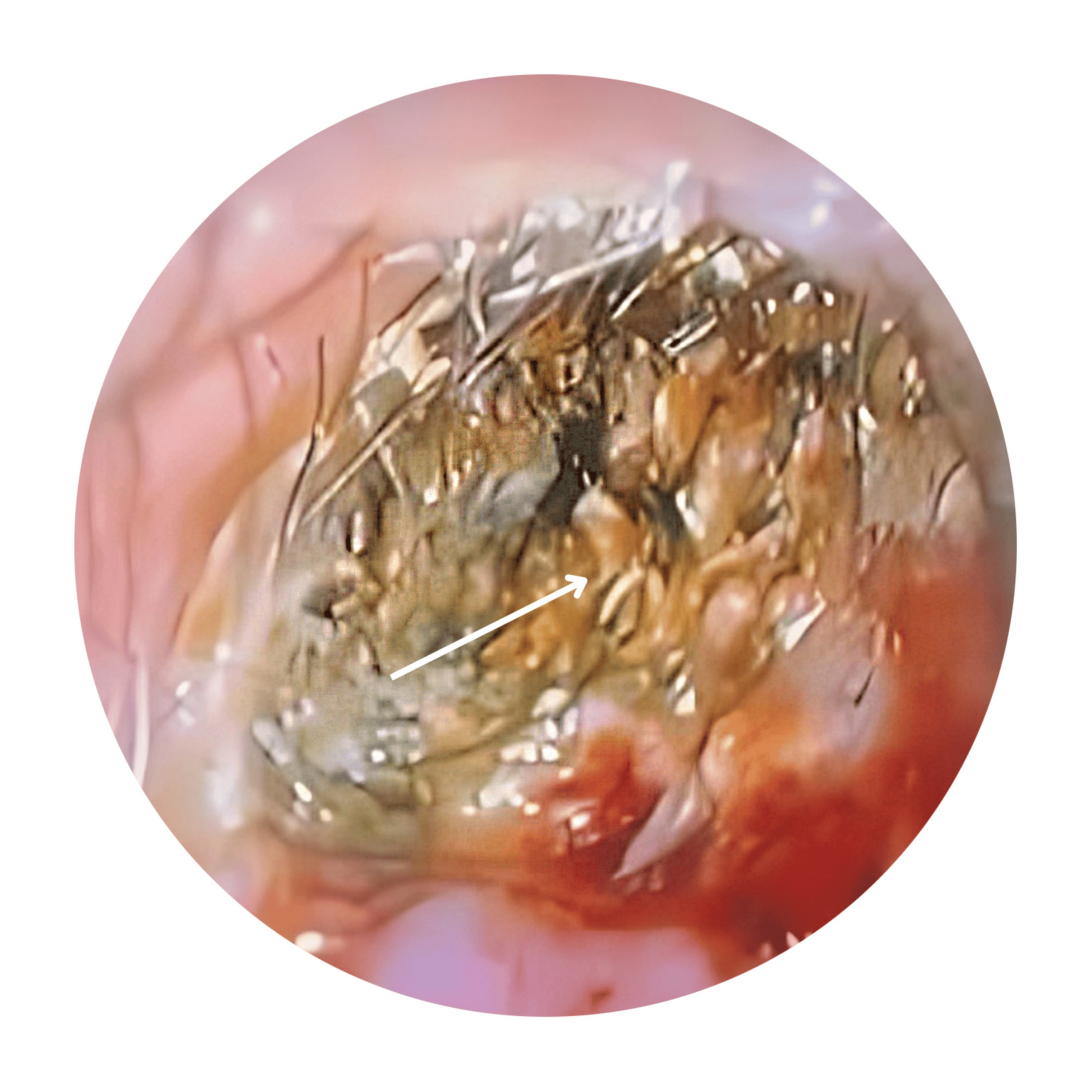
Oto-endoscopic photo of right ear with keratin debris Arrow shows part of the ear canal filled with keratin debris.

An 18-year-old male was referred to our clinic by a general practitioner due to a six-month history of intermittent left otalgia accompanied by reduced hearing. Initially, he had a history of foul-smelling otorrhea, which later subsided, but the ear discomfort and hearing loss persisted. No instances of ear trauma or prior surgery were reported. The patient had sought treatment from multiple general practitioners over the past three months. Otoscopic examination revealed impacted wax with keratin debris. There was cholesteatoma in the external bony canal causing exposed bone with scanty foul-smelling mucopus (Figure 1).

**Figure 2:**
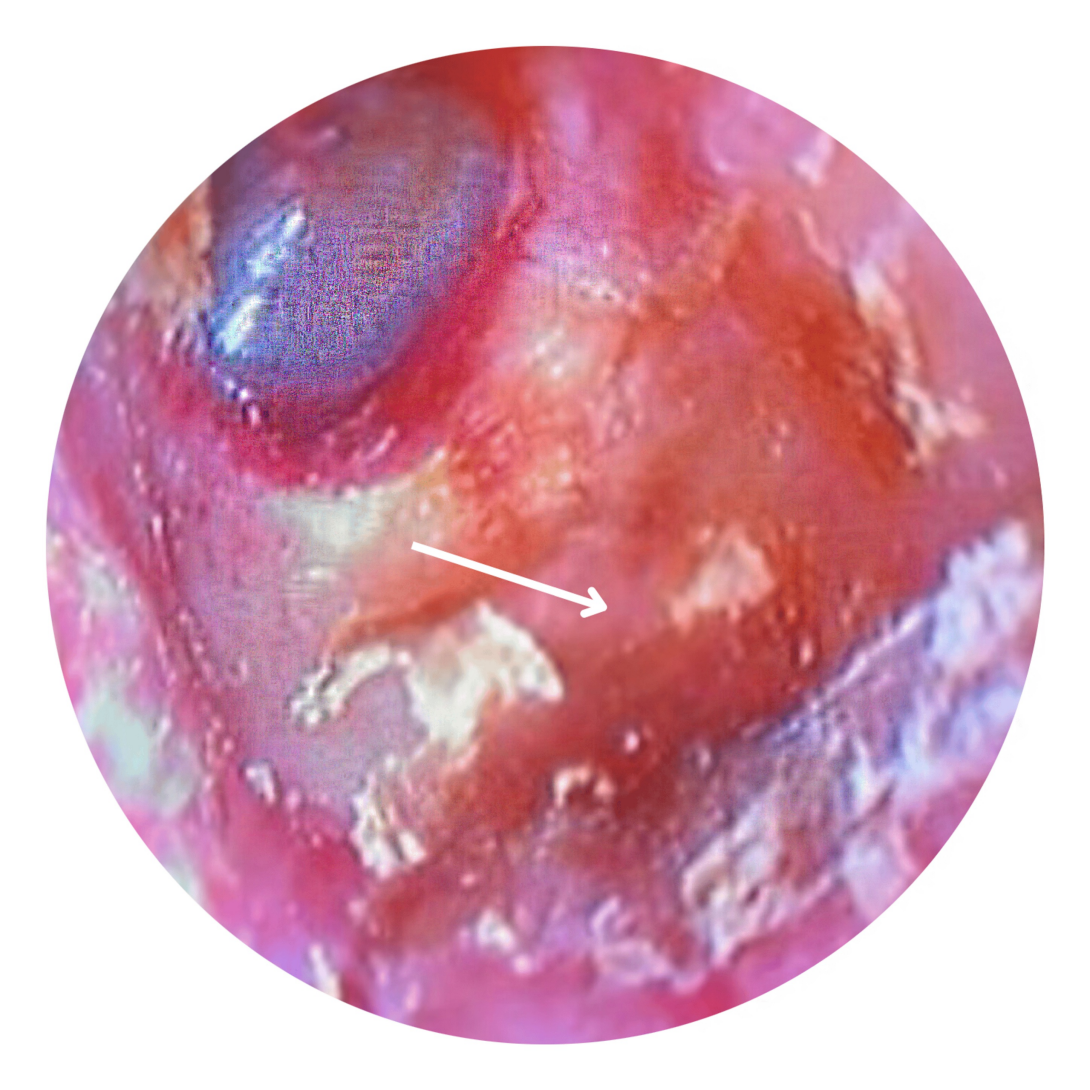
Oto-endoscopic photo of right ear with defect in posterior wall after ear toileting Arrow showed bony erosion over the posterior wall of the ear canal.

The posterior wall and floor were eroded, while the tympanic membrane remained intact (Figure 2). The right otoscopic examination was normal, and no facial palsy was observed. His pure tone audiometry (PTA) reported mild conductive hearing loss in the left ear and normal hearing levels on the right.

**Figure 3:**
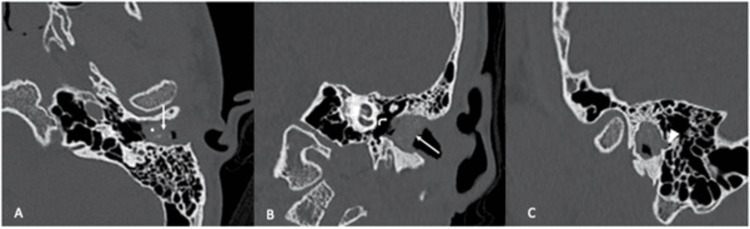
HRCT of the left temporal bone A, B: Axial and coronal images show a soft tissue density (white arrows) with intralesional bone fragments (*) within the left bony EAC. The middle ear cavity is preserved (angled arrow). C: Sagittal image shows focal bone erosion (arrowhead) at the posterior wall of the EAC.

High-resolution computed tomography (HRCT) revealed soft tissue density in the left external ear canal with posterior wall bony erosion, without soft tissue densities observed in the middle ear and mastoid air cells (Figure 3). For this patient, EACC was staged at Stage III according to clinicopathological classification and Stage I according to CT classification [6,7]. The patient was initially scheduled for left canalplasty; however, he opted for a more conservative management approach. Two weekly ear toileting were performed in a clinic setting to remove keratin debris, followed by the application of mupirocin cream. After three months of treatment, his ears were free of cholesteatoma. He continues to attend monthly follow-ups for ongoing ear toileting and monitoring.

## Discussion

Cholesteatoma is a cystic structure composed of stratified squamous epithelium, accumulating desquamated keratin debris, and possessing the potential to erode adjacent structures. While it is more frequently observed in the mastoid and middle ear cavity, its occurrence in the ear canal is rare. According to Im et al. (2020), the estimated incidence of EACC is approximately 6%, with 18 out of 300 ears with congenital aural stenosis developing canal cholesteatoma. In contrast, Casazza et al. (2022) reported an annual cholesteatoma surgery rate of six per 100,000 people in South Korea, which includes cases of canal cholesteatoma [8,9]. Middle-aged to elderly individuals are most commonly affected by EACC, with a study reporting an average age of 41.3 years [3]. However, it can also manifest in younger populations, as exemplified by our case involving an 18-year-old patient.

EACC is characterized by its slow growth as a benign lesion and carries the risk of invasion and damage to surrounding structures. The mechanism behind bone destruction by cholesteatoma varies, with several theories proposed, including pressure theory and enzymatic theory. The pressure theory posits that the growing cholesteatoma applies mechanical pressure on adjacent bone, resulting in bone resorption and necrosis. This is supported by observations that the physical expansion of the cholesteatoma mass exerts direct pressure on the surrounding bone, leading to its destruction [10]. The enzymatic theory highlights the role of biochemical processes, such as enzyme activity and inflammatory mediators, in bone resorption. The presence of biofilms further contributes to the chronicity and persistence of the infection, fostering recurrent inflammation and subsequent bone destruction [11]. Additionally, predisposing factors such as smoking and mechanical aspects like the use of hearing aids and Q-tips have been suggested [5,12]. EACC can occur in patients without any identifiable risk factors, with underlying mechanisms that remain poorly understood. Dubach et al. described this as idiopathic cholesteatoma, emphasizing that the etiology of idiopathic EACC remains unclear and requires further investigation [1]. In our case, the patient had no known risk factors and being in the pediatric age group, presented an atypical occurrence of the condition.

The diagnosis of EACC relies on a thorough patient history and physical examination. Clinical symptoms are nonspecific, commonly presenting as otorrhea, aural fullness, otalgia, hearing loss, and itching. Most of the cases may be asymptomatic, and the symptoms are typically unilateral [13,14]. These manifestations closely resemble those of keratosis obturans and otitis externa, posing a diagnostic challenge, especially for inexperienced general practitioners. Our patient, who presented with unilateral hearing loss and otalgia, reflects these common symptoms.

In general, EACC originates from the inferior wall of the external auditory canal and typically confines itself to the external auditory canal. However, in advanced diseases, erosion may extend inferiorly towards the hypotympanum and jugular dome, anteriorly to the temporomandibular joint, posteriorly into the mastoid, and potentially reaching the vicinity of the facial nerve [5]. Besides common presentation, some of the patients may present as well with facial nerve asymmetry if cholesteatoma has advanced to the facial nerve, trismus, and pain upon chewing if it invades the temporomandibular joint, or pulsatile tinnitus if it invades the jugular dome.

Microscopic ear examination is the initial investigation for EACC. Otoscopy may show an intact tympanic membrane along with localized erosion in the external auditory canal, occasionally associated with impacted cerumen or keratin [1]. Inexperienced practitioners may occasionally misdiagnose the condition as impacted wax or otitis externa. This will obviously lead to incorrect management and worsening EACC. Differential diagnosis for EACC should include keratosis obturans, necrotizing otitis externa, otitis externa, and tumors like ear canal osteoma or squamous cell carcinoma. Other differential diagnoses like granulomatous diseases (granulomatosis with polyangiitis, histiocytosis X) can also be considered [14].

High-resolution computed tomography (HRCT) of the temporal bone is considered the imaging of choice for aiding in the diagnosis of EACC [5]. Both HRCT and, in some cases, magnetic resonance imaging (MRI) are employed to evaluate the extent of the condition, involvement of the middle ear, and impact on neurovascular structures and to distinguish it from other ear canal diseases or tumors. A characteristic finding on HRCT for external auditory canal cholesteatoma is the presence of a soft-tissue mass in the external auditory canal, accompanied by adjacent bone erosion and, at times, intramural bone fragments. The erosion may exhibit a smooth or irregular pattern, attributed to osteonecrosis. In spontaneous EACC, this erosion is frequently observed along the inferior wall of the canal, typically at the bony-cartilaginous junction or the sulcus of the ear canal [15].

The management of EACC is contingent upon the severity of symptoms and the extensiveness of the disease. The primary objective of management is to eliminate the disease and restore normal epithelial migration within the canal, thereby preventing disease progression and recurrence [4]. The treatment approach is guided by the stage of the disease, as classified by He’s staging system, which is well-validated and widely used in clinical practice. In Stage I, lesions are confined to the external auditory canal without bone erosion, and treatment typically involves transcanal cholesteatoma removal (TCR). In Stage II, lesions exhibit bone erosion but remain localized to the external auditory canal, where TCR is the primary treatment, with canalplasty performed when necessary. Stage III is characterized by lesion extension into the mastoid or middle ear, requiring TCR in combination with mastoidectomy and canalplasty. In Stage IV, lesions are extensive, involving the mastoid and adjacent structures, necessitating mastoidectomy, canalplasty, and tympanoplasty. Given the potential for recurrence, particularly in advanced stages, regular follow-up is crucial for long-term disease monitoring and management [16].

Complications associated with EACC include facial nerve palsy, invasion of the mastoid cavity, labyrinthine fistula, and ossicular erosion. Patients may present with unilateral facial asymmetry if the facial nerve is involved, tinnitus, vertigo, and disequilibrium if a labyrinthine fistula occurs, or gradual hearing loss and tinnitus if the ossicular chain is affected. These complications can arise when the condition is not accurately diagnosed and promptly treated. It's noteworthy that intracranial complications resulting from primary EACC are exceedingly rare [5,17].

## Conclusions

In summary, EACC is a rare otological disease. Achieving an accurate diagnosis and administering timely treatment are essential to prevent the occurrence of additional complications. The clinical symptoms are nonspecific and often overlap with those of otitis externa, posing a diagnostic challenge, particularly for inexperienced practitioners. It is essential to consider EACC as a differential diagnosis when suspicion of otitis externa arises. Treatment options range from conservative measures to surgical interventions, with rare recurrence observed with regular follow-up.
